# The Limits of Conscious Deception Detection: When Reliance on False Deception Cues Contributes to Inaccurate Judgments

**DOI:** 10.3389/fpsyg.2020.01331

**Published:** 2020-06-19

**Authors:** Mariëlle Stel, Annika Schwarz, Eric van Dijk, Ad van Knippenberg

**Affiliations:** ^1^Department of Psychology of Conflict, Risk & Safety, University of Twente, Enschede, Netherlands; ^2^Department of Social, Economic, and Organisational Psychology, Leiden University, Leiden, Netherlands; ^3^Department of Social and Cultural Psychology, Radboud University Nijmegen, Nijmegen, Netherlands

**Keywords:** deception, trust, non-verbal cues, conscious thought, lie, truth, information processing, intuition

## Abstract

People are generally too trusting, which decreases their ability to detect deceit. This suggests that distrust could enhance our deception detection abilities. Yet, a state of distrust may induce deliberative conscious thought. This mode of thinking has been related to worse complex decision making. Hence, we investigate whether contextual distrust decreases the ability to detect deceit via the stronger reliance on consciously held beliefs about which cues betray deception. In two studies, participants were asked to judge videos of either deceiving or truth telling targets. Contextual distrust was manipulated by asking participants to squint their eyes (distrust) or to round their eyes (trust) while watching the videos. Participants’ judgments of targets being deceptive or truthful were measured (Studies 1 and 2) and they were asked on what basis they made these judgments (Study 2). Results showed that distrust especially hampers the detection of truth, which is partly due to more reliance on false beliefs about deception cues. These results corroborate the idea that deliberative conscious information processing may hinder truth detection, while intuitive information processing may facilitate it.

## Introduction

The ability to detect deceit is an important skill in everyday life. Studies investigating deception in everyday life, however, show that the majority of deceits are not discovered (DePaulo et al., 1996; Vrij, 2000). When, in laboratory studies, people are explicitly asked whether a person is deceiving or not, the unaided accuracy rate of detecting deception is not far above chance level (e.g., see meta-analyses of Bond and DePaulo, 2006; Hartwig and Bond, 2011). This rate can be increased, among others, with situational familiarity and contextual information (Blair et al., 2010; Reinhard et al., 2013b).

One of the assumed reasons for the relatively poor unaided detection rate is that people have the tendency to believe— rather than disbelieve— information that is being presented (i.e., the “truth bias” or ‘truth-default,” McCornack and Parks, 1986; O’Sullivan, 2003; Levine, 2014). As a result, they often (mis)take a lie for truth. The Truth-Default Theory posits that as most communication is honest most of the time, the benefits of believing outweigh the costs of occasional deception (Levine, 2014; Clare and Levine, 2019). Consequently, people can detect truths with greater accuracy than lies (the veracity effect; e.g., Levine et al., 1999). If too much trust stands in the way of deception detection, wouldn’t being distrusted be the antidote for such gullibility? In the current article, we investigate peoples’ ability to detect deception under conditions of induced trust and distrust. As a trusting motivational state may induce intuitive information processing and a distrusting motivational state may induce deliberative conscious information processing, our article contributes to the debate on the existence of (un)conscious deception detection.

### Suspicion, Distrust and Deception Detection

Research consistently showed that increasing suspicion decreases truth-bias (McCornack and Levine, 1990; Stiff et al., 1992; Millar and Millar, 1997; Hubbell et al., 2001). Studies on the effects of suspicion on deception detection accuracy, however, show mixed results. First, research is inconsistent about *whether* there is a relationship between suspicion and detection accuracy. Several studies showed little or no effect of suspicion on detection accuracy (Toris and DePaulo, 1985; Buller et al., 1991; Stiff et al., 1992), whereas other studies do show an effect (Zuckerman et al., 1982; McCornack and Levine, 1990; Burgoon et al., 1994; Levine et al., 1999; Millar and Millar, 1997; Kim and Levine, 2011).

Second, *if* a relationship between suspicion and detecting deception is observed, the results are inconsistent. On the one hand, Zuckerman et al. (1982) observed that more suspicious participants were less accurate at decoding affect. The results of Burgoon et al. (1994) showed that suspicious experts, but not novices, were less accurate in detecting deception. On the other hand, Levine et al. (1999) observed that suspicion increased lie detection accuracy, but observed a curvilinear relationship for truth accuracy that moderate levels of suspicion results in the greatest accuracy. Finally, the results of Millar and Millar (1997) and also of Kim and Levine (2011) showed that the effect of suspicion on detecting deception depended on whether truths or lies were judged. Suspicion decreased truth accuracy, while increasing lie accuracy. This moderation may also explain why studies not making a distinction between lies and truths did not obtain effects. In sum, although the research on suspicion and detection deception show mixed results, it seems to be the case that suspicion often decreases truth detection accuracy, while increasing lie detection accuracy.

Only a few studies on detecting deception focused on the effects of distrust instead of suspicion. The two concepts seem strongly related, but are distinct (Sinaceur, 2010). In a state of suspicion, perceivers are uncertain about another’s motives, whereas in a state of distrust, perceivers have negative expectations about these motives. As a result, suspicious perceivers are more willing to seek information in order to determine whether another’s motives are honest or not than distrusting perceivers (Sinaceur, 2010). So whereas suspicion influences information seeking among perceivers, distrust influences perceiver’s need to deal with a possibly threatening situation. This may affect their deception detection abilities differently.

Carter and Weber (2010) investigated the connection between (dis)trust and the detections of truths and deceits. In their study, they investigated individual differences in dispositional trust. In doing so, they followed up on Yamagishi’s (2001) argument that some people are in general less trusting than others, and that those who tend to distrust others in life (‘low trusters’) forego learning opportunities for deception detection. Because low trusters are less likely to expose themselves to situations in which they can be deceived, they simply are less likely to learn how to recognize deceit. The results obtained by Carter and Weber (2010) indeed were consistent with this reasoning. In their study, participants were shown eight videos of simulated job interviews of which half were completely truthful and half included a variety of lies. The results showed that participants who were dispositionally more trusting were better at detecting deceit than dispositionally distrusting participants.

The Carter and Weber (2010) study provides first evidence that distrust does not necessarily lead to more accuracy in detecting deceit. Note that the main explanation for the dispositional effects of distrust concentrate on the low truster’s avoidance of learning experiences in the past. In addition to these dispositional differences on trust, people also differ across situations in the extent to which they are trusting (Schlenker et al., 1973). So regardless people’s dispositional trust levels, people may be more or less trusting depending on the specific situation and the individuals they encounter in these situations. We argue that this contextual distrust influences the detecting of deception as well, but via a different process: the deliberative processing of the presented information.

### Intuitive vs. Deliberative Information Processing

According to Schul et al. (2008) trust is default and connotes being safe. Distrust signals that the environment is not normal and, as a result, people avoid routine strategies and more carefully scrutinize people’s behavior (see also Mayo, 2015). Signals that a situation is (potentially) threatening foster deliberate conscious processing, whereas signals that a situation is safe foster less effortful processing (e.g., Schwarz, 1990). This line of reasoning was also followed by Posten and Mussweiler (2013) who contained that distrust may induce non-routine information processing, which in their study led to less reliance on stereotypes (see Conway et al., 2018 for a similar line of reasoning in the domain of morality judgments). And more recently, Thommes and Uitdewilligen (2019) argued and showed in a context of team performance that low trust increased people’s motivation to process task information more elaborately.

Taken together, these insights suggest that a distrusting motivational state promotes deliberative conscious information processing, whereas a trusting motivational state promotes intuitive information processing. Deliberative information processing (i.e., conscious thought) can be defined as controlled and effortful object- or task-relevant cognitive and/or affective thought processes, whereas intuitive information processing can be defined as spontaneous cognitive and/or affective thought processes without intentional effort (e.g., Petty and Cacioppo, 1986; Kahneman, 2003; Strack and Deutsch, 2004). Intuitive decision making may benefit from unconscious thought, defined as thought processes occurring while one’s attention is directed elsewhere to distract from conscious goal-directed deliberation (e.g., Dijksterhuis and Nordgren, 2006). Intuitive decision-making includes both immediate and automatic decisions as well as unconscious thought decisions. Decisions for both forms rely on spontaneous thought processes without intentional effort. The difference between these two forms is that for unconscious thought decisions, the attention is directed elsewhere before making the decision, whereas for automatic decisions, the decision is immediate.

According to Unconscious Thought Theory (Dijksterhuis and Nordgren, 2006), the modes of thinking (deliberative vs. intuitive) differ in their characteristics, which makes each mode preferable in different contexts. First, the conscious mind has difficulties processing large amounts of information. Our conscious capacity is limited: it can temporarily store about 7 items (Miller, 1956). As people consciously can only focus on a limited number of attributes, this goes at the expense of other (relevant) attributes (Wilson and Schooler, 1991). Unconscious thought is assumed to have more processing capacity (e.g., Dijksterhuis, 2004). For instance, Dijksterhuis (2004) showed that unconscious thought leads to better organization and clustering of information in memory. In addition to differences in the amount of information that can be processed, another difference between the two modes of thinking is that conscious thought—compared with unconscious thought—has more difficulties weighing information cues. This is because conscious thought relies more on top-down processing routines, such as rules of thumb and stereotypes (e.g., Bos and Dijksterhuis, 2011). Also, conscious decision-making is affected by contextual influences and consciously held false beliefs (e.g., Reinhard et al., 2013a). Because of these differences in characteristics, complex decisions may be made better without than with conscious thought.

Ample studies on consumer decisions, impression formation, attitude formation, and creativity have indeed demonstrated that complex decisions made with unconscious thought are better than complex decisions made with conscious thought (also termed Unconscious Thought Effect, UTE; for an overview see Dijksterhuis and Nordgren, 2006). However, there are also studies showing that unconscious thought does not (always) lead to better complex decisions (e.g., Acker, 2008; Payne et al., 2008; Calvillo and Penaloza, 2009; Lassiter et al., 2009; Newell et al., 2009; Rey et al., 2009; Thorsteinson and Withrow, 2009; Waroquier et al., 2009, 2010). A meta-analysis including all available published and unpublished data on UTE (Strick et al., 2011) provides support for the validity of UTE, but also identifies boundary conditions and moderators of the effect (e.g., type of distraction task, global vs. specific goal, presentation format). A more recent meta-analysis of Nieuwenstein et al. (2015), however, suggest that such a moderator account of the UTE does not fit their meta-analysis and a large scale replication. Their results seem to point to a publication bias. After correcting for this, the UTE allegedly turned non-significant. Also, the meta-analysis showed that previous UTE studies were underpowered. A meta-analysis of Vadillo et al. (2015) using Bayes factor analysis, however, showed that it is not a lack of power, but that most experimental conditions support the null hypothesis.

### (Un)conscious Thought and Deception Detection

Recent findings suggest that conscious processes can hinder the ability to detect deception (e.g., Reinhard et al., 2013a; Street and Vadillo, 2016). Judging whether a person is truthful or deceptive can be regarded as a complex decision. First, assessing cues, such as level of detail and plausibility, is cognitively demanding (e.g., Forrest and Feldman, 2000). Secondly, processing verbal content as well as non-verbal information and attending to a variety of different types of cues that may be observed, is cognitively demanding as well (e.g., Reinhard and Sporer, 2008). As judging whether a person is deceptive is a demanding process, the Unconscious Thought Theory suggests deception detection can be handled better without conscious thought.

In addition, when judgments of (dis)honesty are made consciously, they may be based on incorrect beliefs about what cues may signal deception (Reinhard et al., 2013a). It should be noted that while correct beliefs increase the ability to detect deception under conditions of distrust (Forrest et al., 2004), lay people often mention wrong cues they rely on. Most people believe that liars show more smiling, gaze aversion, eye blinking, illustrators (i.e., gestures supporting speech), and a more active body, but in reality liars and truth tellers do not differ in these respects (Ekman, 1989; Vrij, 2000; Mann et al., 2002; DePaulo et al., 2003; Sporer and Schwandt, 2007; Hartwig and Bond, 2011). For the present purpose, we refer to these non-verbal cues with the term ‘false deception indicators.’ Although in reality cues of deception are subtle (Hartwig and Bond, 2011; Levine, 2019b), cues that are diagnostic of deception are fewer hand movements, appearing more nervous and having to think hard, being less plausible and less logically consistent, having a higher pitch of voice and using fewer details in a story (Vrij, 2000; DePaulo et al., 2003; Sporer and Schwandt, 2007; Hartwig and Bond, 2011). Note that these diagnostic indicators may be influenced by publication bias (Luke, 2019).

Research indicative that unconscious deception detection may outperform conscious thought showed that indirect measures for deception detection (e.g., “Does the person have to think hard?”) are more accurate than direct measures (“Do you think the person is lying?”; e.g., DePaulo et al., 1997; Vrij et al., 2001; Ten Brinke et al., 2014). The claims that indirect measures outperform direct measures, however, are sometimes based on incomparable metrics (Levine and Bond, 2014; Franz and von Luxburg, 2015; Street and Vadillo, 2016; Levine, 2019b). Meta-analysis showed that most indirect measures do not outperform direct measures (Bond et al., 2014). According to Street and Vadillo (2016), the indirect measures, which do outperform direct measures, may draw attention to the correct indicators of lying. As such, the differential findings for direct versus indirect detection deception methods can be explained without needing unconscious processes.

Indirect evidence that conscious thought can be very successful as well, comes from recent studies that successfully improved the detection of deception with mindful and deliberate strategies, such as inducing situational familiarity, strategic questioning, and strategic use of evidence (for reviews see Levine, 2015, 2019a). This does not imply, however, that unconsciously thinking about the obtained information may not further increase the detection of deception. The information processed consciously as well as unconsciously may trigger people to abandon the default truth-response (for more information about the triggers, see Levine, 2014).

Research directly investigating (un)conscious thought showed that peoples’ ability to detect deception increased when they were kept from consciously deliberating about the presented information (Reinhard et al., 2013a). This increase can be explained by a better integration of diagnostic information: When deciding about the veracity of the presented information, participants relied on *more* information cues in an unconscious mode of thinking compared to conscious thought. Also, the cues were more *diagnostic* of deception. This is in line with research of Vrij et al. (2001) showing that observers rely more on incorrect indicators when they are actively trying to assess whether someone is lying. The findings of Reinhard et al. (2013a) were, however, not reproduced by a high-powered replication study (Moi and Shanks, 2015). As the original UTE, the unconscious deception detection effects may be bound to certain conditions and moderated by several factors (see also Levine, 2019b).

As the evidence for UTE and (un)conscious deception detection is mixed, researchers agree that more research is necessary (e.g., Vadillo et al., 2015; Street and Vadillo, 2016). If (un)conscious thought effects for deception detection exist, the following hypothesis can be formulated with regard to (dis)trust: observers are less accurate in judging whether someone is deceiving or telling the truth under conditions of contextual distrust than under conditions of contextual trust. More specifically, as distrust may induce conscious thought and conscious thought leads to worse deception detection abilities, it is expected that truth tellers will be judged as more deceptive than liars. As trust may elicit intuitive processes, but not necessarily unconscious thought, it is expected that liars and truth tellers will not be distinguished under conditions of trust. So, in our studies we do not test effects of unconscious thought, but compare deception detection abilities when processing information deliberatively versus intuitively.

To test our hypothesis, (dis)trust was varied using a disguised manipulation by having observers adopt facial expressions in line with either distrust –squinted eyes– or trust –wide open eyes. We used this manipulation because previous research and theorizing has demonstrated that squinted eyes are associated with distrust and wide eyes with trust (Zebrowitz, 1997; Schul et al., 2004; Lee and Anderson, 2017). Lee and Anderson, for example, argued that the eyes communicate complex mental states and showed that people associate narrowed eyes with a suspicious mindset. In their discussion, they speculated that these findings could also extend to the persons expressing these eye expressions, which would mean that narrowing the eyes would activate distrust and rounding them would activate trust. Such an account would fit with embodiment theory (Niedenthal, 2007). In agreement with this notion, participants in the current studies were instructed to adopt either such squinted or rounded eye positions (henceforth indicated as eye instructions) while watching video fragments of liars and truth-tellers. Then, they judged the extent to which they thought the person on the video was telling the truth or lying and indicated on which cues they based their judgment. The data of the reported studies are available via the Open Science Framework^1^.

## Study 1

### Methods

This study was carried out in accordance with the American Psychological Association guidelines and was approved by the Ethics Committee of the University of Twente in Enschede, the Netherlands (BCE16176). We report all data exclusions (if any), all manipulations, and all measures in the study. Sample size was a result of terminating data collection after 3 weeks (as was decided beforehand). All subjects gave written informed consent in accordance with the Declaration of Helsinki.

#### Participants and Design

Ninety-three students from University of Twente (62 women and 31 men; *M*_age_ = 25.23, *SD*_age_ = 9.29; age range 18–54 years) participated for course credit points. They were randomly assigned to the conditions of a 2 (context: distrust vs. trust) × 2 (target: liar vs. truth-teller) factorial design with context as between participants variable and target as within participants variable.

#### Procedure

Participants watched eight video fragments displaying a target person who was either lying or telling the truth (see section “Materials”). Before watching the fragment, participants were given eye instructions to manipulate (dis)trust, which they carried out while they watched the video fragment. To disguise the purpose of the manipulation, participants were told that we were interested in the effect of an extra task (the eye instructions) when watching others. One group of participants received instructions to narrow their eyes while watching the fragment, another group to round their eyes. During the study, the experimenter was present to check whether all participants carried out the eye instructions. All participants did so.

The eye instructions are based on Schul et al. (2004) who used facial stimuli with narrowed and rounded eyes to manipulate distrusting and trusting facial expressions (see also Zebrowitz, 1997; Lee and Anderson, 2017). As considering other people’s faces with narrowed eyes as untrustworthy is not the same as the proposition that narrowing one’s own eyes would induce oneself to become less trusting, we pre-tested our instructions in a pilot study (*N* = 24). Participants carried out the eye instructions described above while judging seven pictures displaying different persons. After each picture, they indicated to what extent they felt that the person could be trusted on a 7-points scale (1 = *not at all*; 7 = *very much*). This pilot study demonstrated that narrowing the eyes led participants to trust people less (*M* = 3.90; *SD* = 1.15) than rounding the eyes (*M* = 4.64, *SD* = 0.41), *F*(1,22) = 4.75, *p* = 0.04, $\eta_{\text{p}}^{2}$ = 0.18.

After each video fragment, we measured participants’ deception judgments by asking them on two separate 7-point scales (1 = *not at all*; 7 = *very much*) to what extent they thought the person was telling the truth or was lying (as in Stel and Van Dijk, 2018, Study 2). We measured these deception judgments on a 7-point scale because such measurements may be more sensitive than dichotomous measurements, and because we reasoned that deception judgments such as these are not binary in nature. It should be noted, however, that prior research suggests that the type of measurement generally does not affect deception detection results (Levine et al., 2010).

For exploratory purposes, trust for the person on the video and dispositional distrust were also measured. Trust for the person on the video was measured by asking participants on a 7-point scale (1 = *not at all*, 7 = *completely*) whether they trusted the person on the video. Cronbach’s alpha for the lie videos was 0.38 and for the truth videos 0.59.

Dispositional trust was measured using the General Trust Scale (Yamagishi and Yamagishi, 1994). This questionnaire consists of six items. Three of these items are related to the “belief that [others are] benevolent [persons]” and the other three are related to the “belief that caution is needed in dealing with others.” The items could be answered on a scale from 1 to 5, with 1 implying the lowest level of agreement, and 5 implying the highest level of agreement. Cronbach’s alpha was 0.60. Because of these unacceptable low alpha’s, trust for the person and dispositional trust were not further analyzed^2^.

Finally, participants reported their gender, age, and nationality. At the end, participants were thanked and debriefed.

#### Materials

The videos were developed using a high stakes mock crime paradigm and were produced by Ten Brinke et al. (2014). Targets on the video were instructed to either steal or not steal $100 dollar from an envelope placed between books in the experiment room. They were instructed to convince the experimenter they had not stolen the money (regardless of whether they actually stole the money). The experimenter interrogated the suspects by asking a series of questions, starting with baseline questions (e.g., “What are you wearing today?” and “How is the weather like outside today?”) followed by pleading questions (“Did you steal the money?”, “Why should I believe you?” and “Are you lying to me now?”. The videos were about one and a half minutes long. The eight videos displayed truth and lies, as well as targets’ gender equally.

### Results and Discussion

The measurements of the questions regarding telling the truth and lying were averaged to constitute a ‘deception judgment’ score, after reversing the truth-question, because of their highly correlation (0.89). Deception judgments of truthful and lying targets were normally distributed (skewness scores liars = 0.03; truth tellers = 0.17).

To investigate our contextual distrust hypothesis that observers are less accurate in judging whether someone is deceiving or telling the truth under conditions of contextual distrust than under conditions of contextual trust, a 2 (context: distrust vs. trust) × 2 (target: liar vs. truth-teller) repeated measures analysis of variance (ANOVA) with context as independent variable and with deception judgment of liars and truth tellers as within participants dependent variable was conducted. Table 1 presents means and contrast tests. The analysis yielded the hypothesized interaction between context and target, *F*(1,91) = 46.91, *p* < 0.001, $\eta_{\text{p}}^{2}$ = 0.34. When distrust was activated, participants inaccurately judged truth-tellers as more deceitful (*M* = 4.15, *SD* = 1.12) than liars (*M* = 3.27, *SD* = 0.86), *p* < 0.001, Cohen’s *d* = 0.89. When trust was activated, participants accurately judged liars (*M* = 4.22, *SD* = 0.88) as more deceitful than truth-tellers (*M* = 3.27, *SD* = 0.92), *p* < 0.001, Cohen’s *d* = 1.07. Also, participants judged truth tellers as more deceitful in the distrust than in the trust condition, *p* < 0.001, Cohen’s *d* = 0.87; whereas participants judged liars as more deceitful in the trust than in the distrust condition, *p* < 0.001, Cohen’s *d* = 1.10. There were no main effects of context or target, *F*s < 1.

**TABLE 1:** Means and standard deviations of participants’ judgments of the targets’ deception, as a function of context and target for Study 1 (the higher the scores, the more deceitful, 1 = totally not deceitful, 7 = very much deceitful).

**Context**	**Target**			
	**Liar**		**Truth teller**	
	***M***	***SD***	***M***	***SD***
Distrust	3.27_a_	0.86	4.15_b_	1.12
Trust	4.22_c_	0.88	3.27_d_	0.92

**Means with non-common subscripts differ significantly (p < 0.05) within each column and row.**

## Study 2

The findings of Study 1 showed that induced distrust led participants to inaccurately judge truth tellers to be more deceptive than liars, whereas induced trust led participants to accurately judge liars to be more deceptive than truth tellers. In Study 2, we investigated this effect using different videos. Also, we included a control condition to examine whether distrust hampers the ability to detect deception and/or trust facilitates this.

A second aim of this study was to investigate whether the reliance on the use of false and correct indicators of deception and truth played a role in the effect of (dis)trust on detecting deception. Previous research showed that conscious thought leads people to rely (1) more on false deception indicators (e.g., Vrij et al., 2001) and (2) less on diagnostic cues (Reinhard et al., 2013a). As distrust elicits conscious thought, it is expected that observers in a distrusting state rely more on false indicators of lying and less on diagnostic indicators when judging deception regardless whether targets are deceiving or honest compared to people in a trusting state. We investigate whether reliance on these deception indicators (partly) mediates the effect of contextual (dis)trust on detecting deception.

Finally, to exclude possible alternative explanations of our results due to the used contextual (dis)trust manipulation, we measured participants’ mood and their ratings of the difficulty of the manipulation instructions.

### Methods

#### Participants and Design

Fifty-four students from Leiden University (30 women, 24 men; *M*_age_ = 20.17, *SD*_age_ = 2.69; range_age_: 17–28 years) participated for payment (€2). They were randomly assigned to one of the conditions of a 3 (context: distrust vs. trust vs. control) × 2 (target: liar vs. truth-teller) factorial design.

#### Procedure

The procedure of Study 2 is similar to that of Study 1, except that participants now watched one of four video fragments displaying a target person who was either lying or telling the truth. To investigate whether the effect is generalizable to other videos, we used different videos in the current study (see section “Materials”). As in Study 1, eye instructions while participants watched the video fragment manipulated (dis)trust. In addition to a trust and distrust condition, we added a control condition in which participants did not receive any eye instructions.

After measuring deception judgments (as in Study 1), participants were asked how certain they were of their judgments for exploratory purposes. For exploratory purposes, participants were asked how confident they were of their deception and truth judgments on a 7-point scale (1 = *not at all*; 7 = *very much*).

Then, participants answered an open-ended question on what basis they made their deception and truth judgments. Participants’ responses on the question why they thought the target was lying or telling the truth were rated by counting false and correct indicators of lying. Based on the meta-analyses of DePaulo et al. (2003), Sporer and Schwandt (2007), and Hartwig and Bond (2011) indicators were classified as false when participants relied on cues that are unreliable indicators of deception or the truth. These are: gaze aversion, eye blinking, smiling, illustrators, movements of the body, posture, and appearance. This also includes incorrectly believing that more hand movements are indicative of lying. Indicators were classified as correct when it was correctly mentioned that the above listed false indicators are indeed non-diagnostic of deception and that fewer hand movements, appearing more nervous, and having to think hard were diagnostic. As these correct beliefs about truth and deception include correct beliefs about non-diagnostic cues as well, we also calculated the number of diagnostic cues separately (i.e., fewer hand movements, appearing more nervous, and having to think hard). The diagnostic cues ‘being less plausible,’ ‘being less logically consistent,’ ‘having a higher pitch of voice,’ and ‘using fewer details in a story’ (DePaulo et al., 2003) were not included as the videos used in Study 2 focused on non-verbal cues only.

Finally, to control for possible unintended effects of the instructions, we included three measures. First, participants’ mood was measured by asking them to rate how tense, enthusiastic, pleased, worried, irritated, angry, confused, cheerful, dreary, happy, and sad they felt. Second, participants rated the difficulty of carrying out the instructions. All questions were answered on a 7-point scale (1 = *not at all*; 7 = *very much*). Third, in an open-ended question about the intended goal of the study none of the participants mentioned a link between the eye instructions and deception detection. Trust judgments for the person on the video and dispositional trust were not included as measures in this study.

#### Materials

The fragments were obtained when unobtrusively recording target persons’ body and face while they lied or told the truth to another student about a film fragment which only the target persons had seen (similar to Stel and Van Dijk, 2018). The target persons believed that the other student was instructed to find out whether they lied or told the truth and it was their job to convince the other student that they were telling the truth. To raise the stakes, we told that the ability to convince others would be relevant to their career as a psychologist and would predict future success. The first minute of each target’s presentation was selected. Afterward, the target persons were debriefed about the real purpose of the study and were asked for permission to use the recorded material. The videos did not display sound as these were made with the purpose to investigate non-verbal behaviors only.

### Results and Discussion

#### Deception Judgment

As in Study 1, the two deception items were averaged to a deception judgment score, after reversing the scores for the truth-item (Cronbach’s alpha = 0.79). Deception judgments of truthful and lying targets were normally distributed (skewness scores liars = 0.33; truth tellers = 0.28).

To investigate the contextual distrust hypothesis that observers are less accurate in judging deception under conditions of contextual distrust than under conditions of contextual trust, a 3 (context: distrust vs. trust vs. control) × 2 (target: liar vs. truth teller) ANOVA with context as independent variable and participants’ deception judgment of the target as a dependent variable was conducted. Table 2 presents means and contrast tests. A main effect of context, *F*(2,48) = 5.21, *p* = 0.01, $\eta_{\text{p}}^{2}$ = 0.18, indicated that participants rated the target as less deceptive when trust was induced (*M* = 2.97, *SD* = 1.14) than when distrust was induced (*M* = 4.25, *SD* = 1.52), *p* = 0.003, Cohen’s *d* = −0.99, and than participants in the control condition (*M* = 3.76, *SD* = 1.25), *p* = 0.05, Cohen’s *d* = −0.68. The distrust and control condition did not differ significantly, *p* = 0.22, Cohen’s *d* = 0.37.

**TABLE 2:** Means and standard deviations of participants’ judgments of the targets’ deception, as a function of context and target for Study 2 (the higher the scores, the more deceitful, 1 = totally not deceitful, 7 = very much deceitful).

**Context**	**Target**			
	**Liar**		**Truth teller**	
	***M***	***SD***	***M***	***SD***
Distrust	3.19_a_	1.00	5.31_b_	1.16
Trust	3.05_a_	1.42	2.89_a_	0.78
Control	3.86_a_	1.03	3.63_a_	1.58

**Means with non-common subscripts differ significantly (p < 0.05) within each column and row.**

A marginally significant main effect of target, *F*(1,48) = 3.13, *p* = 0.08, $\eta_{\text{p}}^{2}$ = 0.06, Cohen’s *d* = 0.40, showed that participants tended to rate the truthful target as more deceptive (*M* = 3.90, *SD* = 1.55) than the lying target (*M* = 3.40, *SD* = 1.19).

Both main effects were qualified by an interaction effect of context and target, *F*(2,48) = 5.39, *p* = 0.01, $\eta_{\text{p}}^{2}$ = 0.18. The truth telling target was rated as more deceptive (*M* = 5.31, *SD* = 1.16) than the lying target (*M* = 3.19, *SD* = 1.00) in the distrust condition only, *p* = 0.001, Cohen’s *d* = 2.09, not in the trust and the control condition, *F*s < 1. Additionally, context only affected judgment ratings for truth telling targets, *p* < 0.001, not for lying targets, *p* = 0.29: Truth telling targets were rated as more deceptive in the distrust condition (*M* = 5.31, *SD* = 1.16) than in the trust condition (*M* = 2.89, *SD* = 0.78), *p* < 0.001, Cohen’s *d* = 2.64, and than in the control condition (*M* = 3.63, *SD* = 1.58), *p* = 0.01, Cohen’s *d* = 1.30. The trust and control condition did not differ significantly, *p* = 0.22, Cohen’s *d* = −0.65.

#### Indicators of Deception

False indicators and the total number of indicators were normally distributed (skewness scores false indicators = 0.10; total number of indicators = 0.13). Correct beliefs (total) and diagnostic cues were not normally distributed (skewness score = 0.74 and 0.72). Therefore, separate tests for false and total number of indicators were conducted with ANOVA’s and for correct beliefs and diagnostic cues with Kruskal–Wallis *H* tests.

##### False indicators

To test our indicator hypothesis that observers in a distrusting state rely more on false indicators of lying when judging deception regardless whether targets are deceiving or honest compared to people in a trusting state, a 3 (context: distrust vs. trust vs. control) × 2 (target: liar vs. truth teller) ANOVA on the number of false indicators was conducted. Table 3 presents means and contrast tests.

**TABLE 3:** Means and standard deviations of participants’ number of mentioned false, correct, and unrelated indicators of lying, as a function of context and target for Study 2.

**Indicators target context**	**False**				**Correct**				**Diagnostic**			
	**Liar**		**Truth teller**		**Liar**		**Truth teller**		**Liar**		**Truth teller**	
	***M***	***SD***	***M***	***SD***	***M***	***SD***	***M***	***SD***	***M***	***SD***	***M***	***SD***
Distrust	0.75_a_	0.71	1.75_b_	0.89	0.88_a_	0.64	0.25_a_	0.46	0.63_a_	0.52	0.25_a_	0.46
Trust	0.90_a_	0.74	1.33_a__b_	0.71	0.70_a_	0.95	0.44_a_	0.53	0.60_a_	0.84	0.44_a_	0.53
Control	1.09_a__c_	0.70	0.50_c_	0.93	0.45_a_	0.52	0.88_a_	0.83	0.45_a_	0.52	0.75_a_	0.71

**Means with non-common subscripts differ significantly (p < 0.05) within each indicator.**

There was no main effect of target, *F*(1,48) = 1.75, *p* = 0.19, $\eta_{\text{p}}^{2}$ = 0.04, nor a main effect of context, *F*(2,48) = 1.60, *p* = 0.21, $\eta_{\text{p}}^{2}$ = 0.06. An interaction effect between context and target, *F*(2,48) = 4.74, *p* = 0.01, $\eta_{\text{p}}^{2}$ = 0.17, indicated that when targets told the truth, participants mentioned more false indicators of lying (*M* = 1.20, *SD* = 0.96) than when the target was lying (*M* = 0.93, *SD* = 0.70) in the distrust condition only, *p* = 0.01, Cohen’s *d* = 0.34; not in the trust and the control condition, *F*s < 1.43, *p*s > 0.16, Cohen’s *d* < 0.78.

##### Correct indicators

To investigate whether participants had more *correct beliefs* about cues for liars and/or truth tellers depending on context, two Kruskal–Wallis *H* tests were conducted for lying and truth telling targets separately. These analyses showed that context did not significantly influence correct indicators of deception for lying targets χ(2)^2^ = 1.66, *p* = 0.44, nor for truth telling targets χ(2)^2^ = 3.12, *p* = 0.21.

To investigate whether participants used more *diagnostic indicators* for deception and the truth depending on context, two Kruskal–Wallis *H* tests were conducted for lying and truth telling targets separately. These analyses showed again that context did not significantly influence correct indicators of deception for lying targets χ(2)^2^ = 0.44, *p* = 0.80, nor for truth telling targets χ(2)^2^ = 2.62, *p* = 0.27.

##### Total number of indicators

To test whether participants used more indicators in total for liars and/or truth tellers depending on context, a 3 (context: distrust vs. trust vs. control) × 2 (target: liar vs. truth teller) ANOVA with total numbers of indicators was conducted. There were no effects of context, target, or an interaction between the two, *F*s < 1 (overall: *M* = 1.65, *SD* = 0.83).

#### Correlations and Mediation

The deception judgment was significantly correlated with the number of false indicators of lying, *r* = 0.41, *n* = 54, *p* = 0.002, and not with correct beliefs, *r* = −0.07, *n* = 54, *p* = 0.60, or diagnostic indicators of lying, *r* = −0.06, *n* = 54, *p* = 0.67.

To test whether the effect of context and target on deception judgments was indeed mediated by the reliance on false indicators, we used the regression method proposed by Baron and Kenny (1986). The context dummy contrasts distrust versus trust and no instruction. First, the interaction effect between context and target on number of false indicators is significant, *B* = −0.27, *t* = −2.22, *p* = 0.03. In a different regression model, the interaction between context and target produced significant effects on deception judgment, *B* = −0.59, *t* = −3.27, *p* = 0.002. After inclusion of the mediator, this interaction effect remained significant, *B* = −0.48, *t* = −2.61, *p* = 0.01. Moreover, the number of false indicators significantly affected the deception judgment, *B* = 0.43, *t* = 2.07, *p* = 0.04. These findings show that the use of false indicators of lying partially mediated the effect of context and target on deception judgments, Sobel: *z* = −2.07, *p* = 0.04.

#### Confidence in Deception Judgment

To explore whether context influenced participants’ confidence in their deception judgment, a 3 (context: distrust vs. trust vs. control) × 2 (target: liar vs. truth teller) ANOVA with participants’ ratings of their certainty in judging deception was conducted. The analysis showed no main or interaction effects, *F*s < 1.

#### Mood

The mood items were combined into the mean of the twelve mood items, after reversing the negative items (Cronbach’s alpha = 0.71). To test whether mood could alternatively explain the results, a 3 (context: distrust vs. trust vs. control) × 2 (target: liar vs. truth teller) ANOVA with participants’ mood as dependent variable was conducted. There was no main effect of context, *F*(2,48) = 2.11, *p* = 0.13, $\eta_{\text{p}}^{2}$ = 0.08, no main effect of target, nor an interaction effect between context and target, *F*s < 1. If anything, the main effect of context showed the pattern that participants in the control condition felt less positive (*M* = 4.44, *SD* = 1.11) than participants distrust condition (*M* = 4.87, *SD* = 0.57), *p* = 0.13. Cohen’s *d* = −0.49, or than participants in trust condition (*M* = 4.96, *SD* = 0.62), *p* = 0.06. Cohen’s *d* = −0.59. The distrust and trust condition did not differ significantly in mood, *p* = 0.74. Cohen’s *d* = −0.16.

#### Difficulty in Carrying Out the Instructions

To test whether differences in difficulty of carrying out the eye instructions could have influenced the results, a 3 (context: distrust vs. trust vs. control) × 2 (target: liar vs. truth teller) ANOVA with participants’ ratings of the difficulty of carrying out the instructions as a dependent variable was conducted. There was no main effect of target, *F* < 1, nor an interaction effect between context and target, *F*(2,48) = 1.22, *p* = 0.31, $\eta_{\text{p}}^{2}$ = 0.05. A main effect of context, *F*(2,48) = 11.24, *p* < 0.001, $\eta_{\text{p}}^{2}$ = 0.32, showed that participants in the control condition rated the instructions as less difficult (*M* = 2.00, *SD* = 1.41) than participants in the distrust condition (*M* = 4.37, *SD* = 1.36), *p* < 0.001, Cohen’s *d* = −1.76, or than participants in the trust condition (*M* = 4.05, *SD* = 2.09), *p* < 0.001, Cohen’s *d* = −1.18. The distrust and trust condition did not significantly differ in difficulty, *p* = 0.59, Cohen’s *d* = 0.18.

So importantly, the obtained results on detecting deception and the reliance on false indicators cannot be explained by mood or difficulty in carrying out the instructions.

## General Discussion

Although it seems evident to expect that enhancing distrust should reduce the truth-bias that people tend to believe others in general, the results did not show that distrusting people were less likely to (mis)take a lie for the truth. On the contrary, our findings indicate that distrust led participants to hold truths for lies. More specifically, the results of Studies 1 and 2 showed that contextual distrust led participants to inaccurately judge truth tellers as more deceptive than liars. Furthermore, the results of Study 2 showed that people who were distrusting relied more on false beliefs about lying when judging truth tellers than when judging liars.

Importantly, induced distrust did not lead participants to conclude that all targets were deceitful. This is in line with the notion that inducing contextual distrust may elicit a specific mode of thinking: deliberative conscious information processing. The finding that a distrusting state led participants to rely more on their conscious but false beliefs about cues of deception would fit such an account. Moreover, the finding that a conscious state of mind hampers detecting deception and increases the use of more incorrect indicators, is in line with the results of Vrij et al. (2001) and Reinhard et al. (2013a). Although we did not directly test the existence or benefits of unconscious deception judgments, we add to this debate by showing that contextually induced modes of thinking affect the ability to detect deception.

### Detecting Deception

Previous research showed that *dispositional* distrust leads to worse detection of deception allegedly due to lack of experience with deceptive situations (Carter and Weber, 2010). The current research adds that *contextual distrust* hampers the ability to detect lies, via a different process: due to more reliance on false beliefs about deception, distrusting observers are less able to detect truths. The results imply that although people might be generally trusting or distrusting, encountering a situation which induces distrust leads them to rely more on false beliefs of deception and to be less accurate in detecting truths. One might argue that because dispositionally trusting individuals have more experience with deceptive situations, they are less likely to rely on these false beliefs about lying, and thus the effects should be less strong. Previous research, however, showed that experienced people (police officers) also hold these false beliefs about deception (Vrij et al., 2001).

The main result that contextual distrust led participants to inaccurately judge truth tellers as more deceptive than liars is due to truth tellers being seen as more deceptive (when compared to the control condition). Previous research on suspicion, even though mixed, seems to suggest that suspicion decreases truth detection accuracy and increases lie detection accuracy. Our findings are partly in line by showing that distrust decreases truth detection accuracy. That distrust did not increase lie detection accuracy, whereas suspicion generally does, may reflect differences in the concepts of distrust and suspicion (Sinaceur, 2010). When being suspicious, people will seek more information regardless whether the person is lying or telling the truth. When being distrusting, people already have negative expectations, this is why distrust may not have increased lie detection accuracy. Our results showed that participants in the distrust condition relied more on their conscious false beliefs about deception cues when judging truth tellers.

### Indicators of Deception

We found that reliance on false indicators decreased deception detection, not that correct indicators increased this. It is not in line with our expectations and with previous research that reliance on diagnostic cues is unrelated to accuracy (e.g., Hartwig and Bond, 2011). We do think, however, that our results are valuable by showing that using diagnostic cues does not always lead to better detection of deception. First, it is possible that participants relied on other cues as well which they did not report (see also Hartwig and Bond, 2011). Second, a moderating factor may be the type of videos we used (non-verbal only). Because we used videos focusing on non-verbal cues only, the diagnostic cues participants could rely on were reduced. As multiple cues increase diagnostic value (Hartwig and Bond, 2014), it is conceivable that the number of diagnostic cues displayed moderates the effect of diagnostic cues on the ability to detect deception. A meta-analysis on all data sets, including those who also did not obtain a relationship between diagnostic cues and detecting deception (see also Luke, 2019) may shed light on possible moderators.

Importantly, we do show that reliance on false cues of deception decreases the ability to detect deception. These results are in line with Vrij et al. (2001) who showed that participants who (partly) relied on non-diagnostic cues (in the direct measurement condition) were worse at detecting deception than participants who relied on diagnostic cues only (in the indirect measurement condition). We did not expect, however, that within the distrust condition the reliance on false indicators would differ for liars and truth tellers. This result seems puzzling as when participants would use uninformative cues only, they would not respond differently to deceivers and truth tellers. The category of false indicators, however, includes incorrectly mentioning more hand movements for deceivers and fewer hand movements for truth tellers, whereas in reality, fewer hand movements are indicative of liars (Sporer and Schwandt, 2007; Hartwig and Bond, 2011). This may explain why the use of these false indicators of liars when judging truth tellers in the distrust condition leads to worse deception judgments compared with truth tellers in the trust and control condition.

This is related to the strategies used by deceivers and truth tellers. Even though both liars and truth tellers would like to appear credible, liars worry about giving away that they are lying via non-verbal and verbal cues. As such, liars often try to control their behavior (Hamlin et al., 2020). Truth tellers, on the other hand, are nervous as well, but as they do not have to worry about leaking cues of deception, they do not control their movements. This may explain why liars show fewer hand movements compared with truth tellers, opposite of what observers may expect and, consequently, why we found that making use of this incorrect cue of deception leads to worse deception judgments when judging truth tellers compared with liars. This effect only occurred when distrusting a source, as observers more strongly relied on these false beliefs and, as a result, take a truth for a lie. This way, being distrustful ironically hampers one’s ability for accurate truth detection.

An important factor is that distrusting observers are liable to use the wrong deception cues. However, the reliance on these wrong cues only partially mediated the contextual distrust results on detecting deception. In line with previous arguments (e.g., Dijksterhuis, 2004; Reinhard et al., 2013a), we suggest that the results may additionally reflect that distrusting participants are less capable of organizing and integrating the presented information as a distrusting state induces deliberative information processing. It would be interesting to test this idea in future research.

### (Un)conscious Thought

The results that induced distrust led to worse deception judgments compared with induced trust, fit with the notion of the Unconscious Thought Theory that complex decisions are better made without conscious thought. The finding that contextual trust did not induce participants to use more correct indicators does not directly support the idea that unconscious thought leads to better complex decision-making (e.g., Dijksterhuis, 2004). Note, however, that our contextual trust condition is assumed to elicit intuitive information processing, not to facilitate unconscious thought specifically. Our participants were not given much time to unconsciously think about the decision, and were also not distracted in between the presentation of the information and the judgment. Nevertheless, in Study 1 we did find that induced trust led participants to accurately distinguish between liars and truth tellers. These findings were not replicated in Study 2, although truth-telling targets were rated more accurately in the trust than in the distrust condition. So these results imply that a context eliciting intuitive information processing without unconscious thought does not lead people to rely on more or on more correct use of indicators. Such a context, however, does facilitate truth detection.

The differences in findings that trust leads to more accurate deception detection in Study 1, but only partly (for truth tellers only) in Study 2, may be due to the different videos we used in Studies 1 and 2. In Study 1, we used videos in which liars may be better detected due to the interrogative nature of the video which started by asking all targets baseline questions on which they answered truthfully. The assumption is that it is easier to discover lying behavior of a person when people are familiar with truthful behavior of this same person, due to a change in the person’s non-verbal behavior (e.g., Vrij and Mann, 2001; Mann et al., 2002; Porter and Ten Brinke, 2010). On the videos of Study 2, liars displayed deceptive behavior only and non-verbal behavior only, which makes it harder to detect the liars of Study 2 than of Study 1. The difference in the results of both studies seems to suggest that, depending on the way the information is presented or asked for, intuitive information processing may facilitate detecting liars as well.

### Limitations

A limitation of our research is that the sample size of Study 2 is low (*N* = 54). As such, the results should be interpreted with caution. Importantly, our main finding that when distrust was activated, participants inaccurately judged truth-tellers as more deceitful than liars was obtained with a larger sample in Study 1 (*N* = 93) in which target was a within subject variable. In both studies, the obtained interaction effect of context and target on the deception judgment had a large effect size ($\eta_{\text{p}}^{2}$ of Study 1 = 0.34; $\eta_{\text{p}}^{2}$ of Study 2 = 0.18; Cohen, 1988). Also the specific finding within the distrust condition that participants judged truth-tellers as more deceitful than liars had a large effect in both studies (Cohen’s *d* of Study 1 = 0.89; Cohen’s *d* of Study 2 = 2.09). The additional analyses on indicators of deception were, however, only investigated in Study 2. These should be interpreted with caution. Although the main interaction effect of context and target on false indicators was a large effect ($\eta_{\text{p}}^{2}$ = 0.17), the specific comparison within the distrust condition that participants relied more on false indicators of lying when judging truth-tellers compared to liars had a small to medium effect size (Cohen’s *d* = 0.34).

In our studies, we did not directly manipulate (un)conscious thought. Our argument is that distrust induces deliberative processing as distrust signals that a problematic situation exists, which fosters deliberation (e.g., Schwarz, 1990). Previous research also showed that distrust leads people to more elaborately process information and more carefully scrutinize people’s behavior, which reflects deliberation (e.g., Schul et al., 2008; Posten and Mussweiler, 2013; Conway et al., 2018; Thommes and Uitdewilligen, 2019). Nevertheless, future research should test our proposed mechanism behind the effect, i.e., that the effect is indeed caused by (dis)trusting states influencing intuitive or deliberative thought.

Another limitation of our studies is that we induced contextual (dis)trust by a subtle eyes-manipulation, not by situational or environmental cues. Even though manipulating distrust via the (social) environment would have created a more real life situation, a subtle and unobtrusive manipulation unrelated to the information presented as used in the present studies is less prone to alternative interpretations of the results. While we do not have any reason to expect that contextual distrust as elicited in a specific (social) environment would not show similar effects, it would be interesting to test this in future research.

## Conclusion

We showed that contextual distrust hampers peoples’ ability to detect deception especially for truth tellers, partly because people rely more on their false beliefs about deception. Also, depending on the way the information is presented, contextual trust facilitates the detecting of deception. As a distrusting motivational state fosters deliberative information processing and a trusting motivational state fosters intuitive information processing, these insights contribute to the debate on the existence of (un)conscious deception detection: deliberative conscious information processing hinders the ability to detect deception, while intuitive information processing is beneficial, at least when it comes to detecting the truth. Investigating contexts that differ in modes of processing provides a new way of investigating the existence of (un)conscious deception detection.

## Data Availability Statement

All datasets generated for this study are included in the article/supplementary material.

## Ethics Statement

The studies involving human participants were reviewed and approved by BMS Ethics Committee, University of Twente. Written informed consent from the participants’ legal guardian/next of kin was not required to participate in this study in accordance with the national legislation and the institutional requirements.

## Author Contributions

MS, AS, ED, and AK contributed to the study concept and design. AS performed the data collection of Study 1. MS performed the data collection of the pilot study and Study 2, data analyses and interpretation, and drafted the manuscript. ED and AK provided critical revisions. All authors approved the final version of the manuscript for submission.

## Conflict of Interest

The authors declare that the research was conducted in the absence of any commercial or financial relationships that could be construed as a potential conflict of interest.

## Footnotes

1 https://osf.io/5xyts/

2 Moreover, the measure of trust for the person of the video seem conflated. Given our setup, in which the question to what extent they trust the person on the video was asked after the veracity judgment, the measurement was probably more indicative of whether participants felt the person was speaking the truth than of general distrust.
